# Supplementary figures and images for: Structural and evolutionary characteristics of dynamin-related GTPase OPA1
Source: PeerJ. 2019 Jul 8;7:e7285. doi: 10.7717/peerj.7285 (PMC6622160; doi:10.7717/peerj.7285)

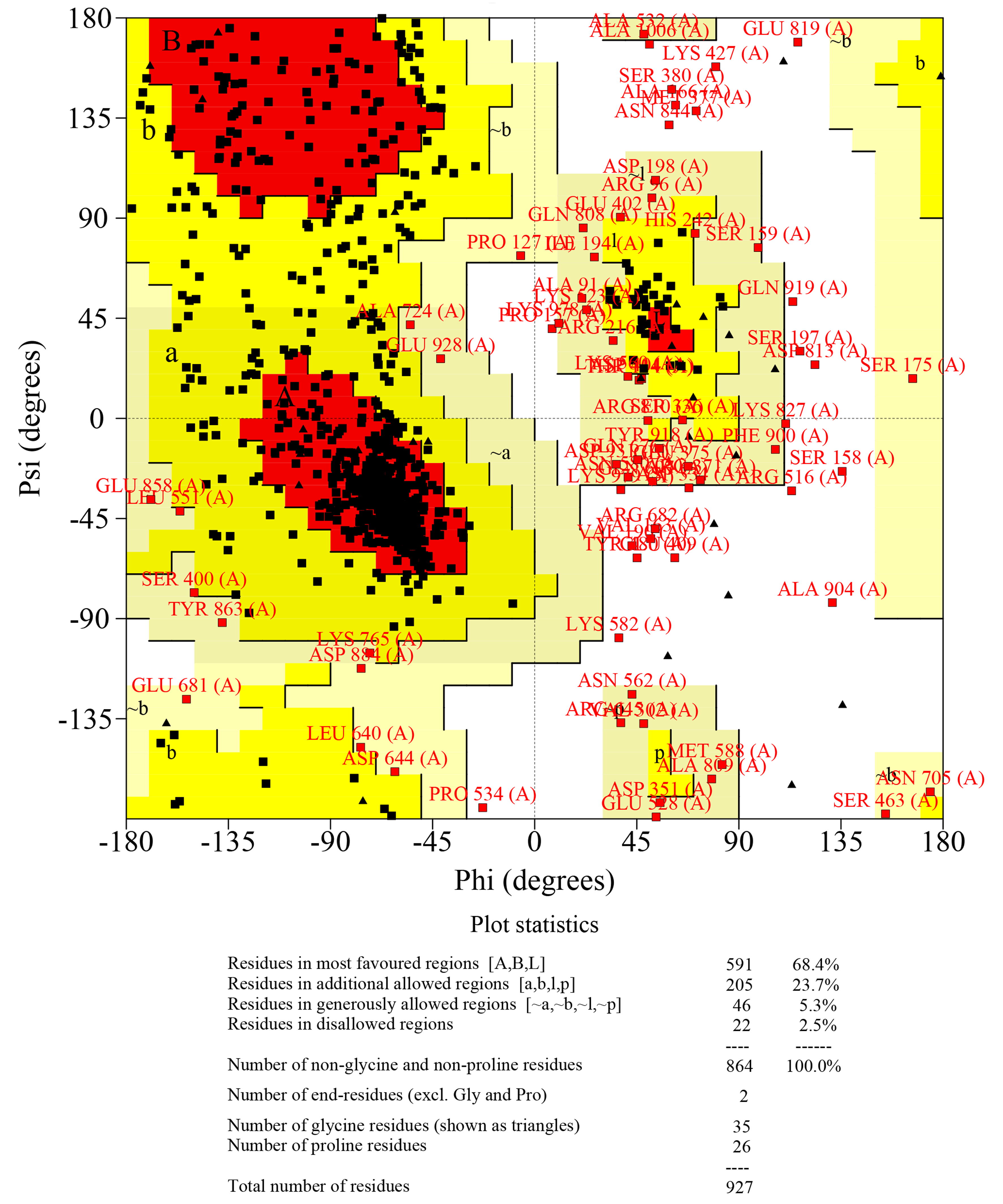

Supplement: Figure S1 — The statistical data of Ramachandran plot was calculated by PROCHECK. The results showed that the proportion of core% + allow% (the core residues: residues in most favoured regions; allow residues: residues in additional allowed regions) was more than 90% for the protein structure of all predicted OPA1 domains. While, the ratio of all disallowed residues (residues in disallowed regions) was less than 5%. According to quality assessment, the accuracy of the OPA1 protein structural models were acceptable. [file peerj-07-7285-s001.png]

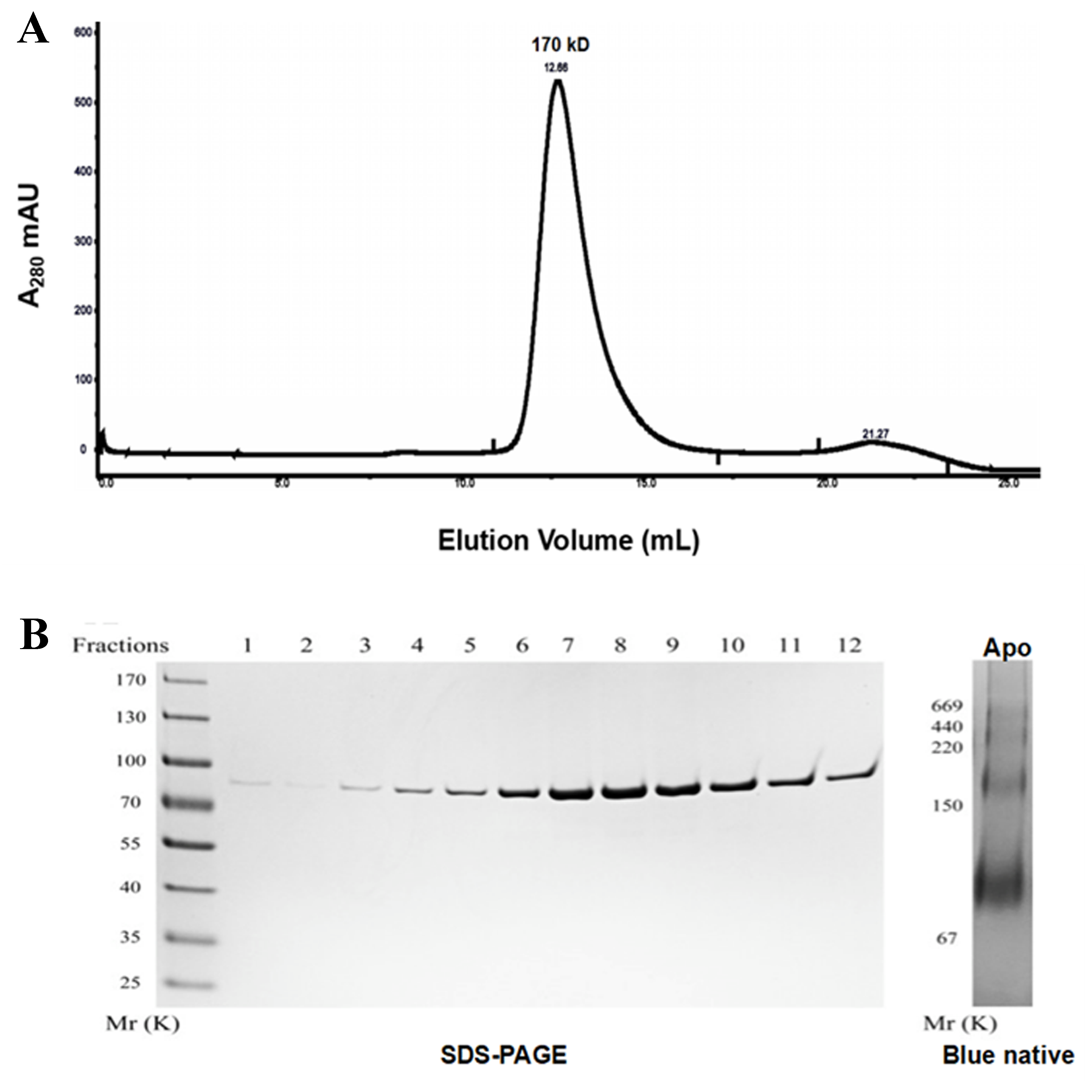

Supplement: Figure S2 — (A) The OPA1 (exons 6-28) elution profiles of a S200 size-exclusion column. (B) SDS-PAGE and Blue native PAGE analysis of purified human OPA1 (exons 6-28) protein. Purification was carried out more than 10 times for OPA1 with similar purity and yield. [file peerj-07-7285-s002.png]

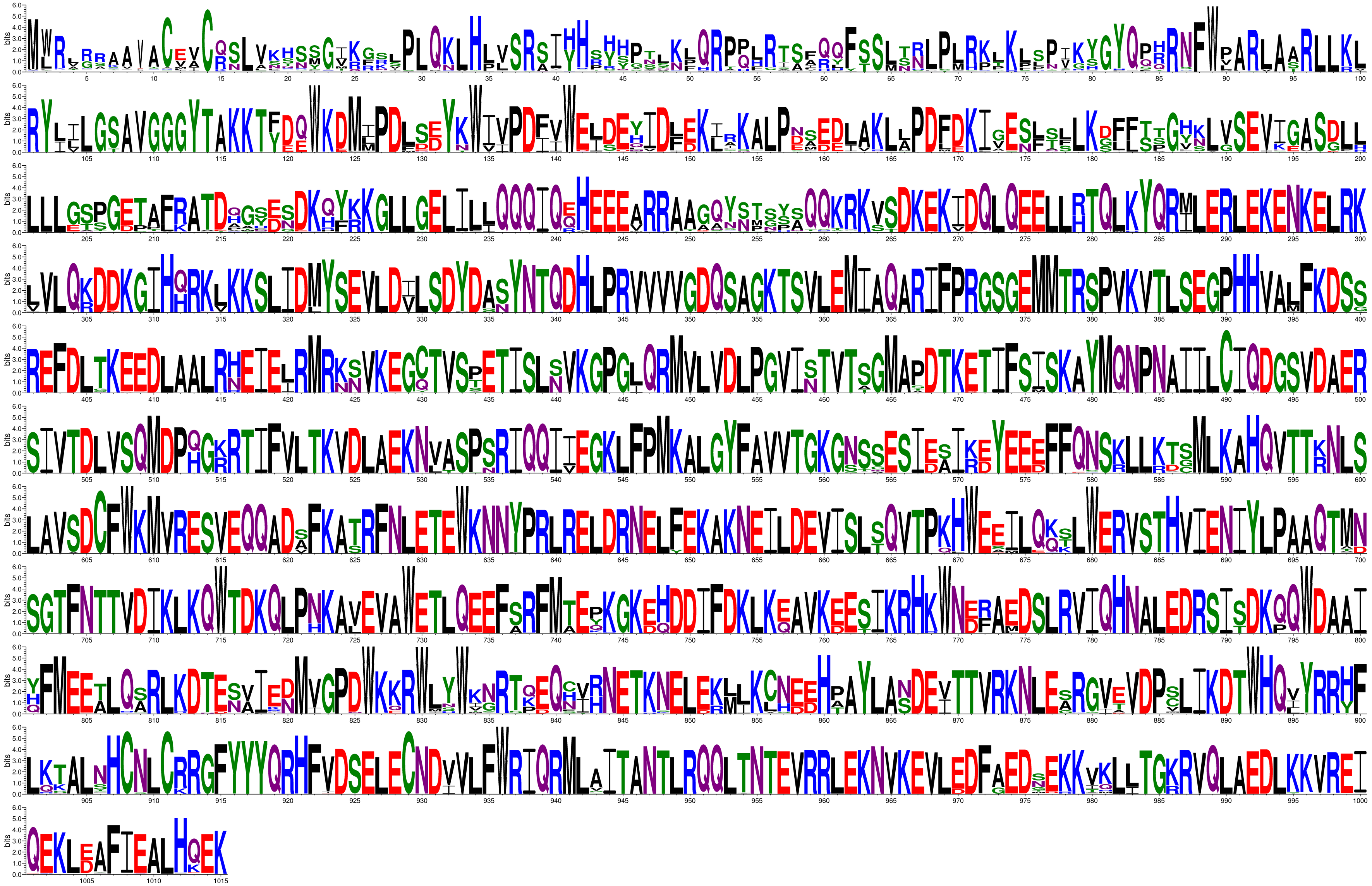

Supplement: Figure S3 — Sequence logos were generated as graphical representations of the multiple sequence alignments of the amino acids. The positions of the amino acid sites in vertebrates OPA1 protein are in accord with those of human OPA1 isoform 8. [file peerj-07-7285-s003.png]
