# Supplementary material for: Structural and evolutionary characteristics of dynamin-related GTPase OPA1
Source: PeerJ. 2019 Jul 8;7:e7285. doi: 10.7717/peerj.7285 (PMC6622160; doi:10.7717/peerj.7285)
Supplement: Data S1 [file peerj-07-7285-s004.docx]

**Supplementary data 1. The information of known disease-related sites of the human OPA1 protein.**

| **Positions^a^** | **Natural variant^b^** | **Diseases^c^** | **Exons**  **(Domain)** | **Conservation**  **(grade)^d^** | **References** |
| --- | --- | --- | --- | --- | --- |
| 4-5 | LR→ - (del) | likely pathogenic | 1-2 | 1, 1 | ([Hayashi et al. 2017](#_ENREF_22)) |
| 8 | A→S (sub) | pathogenic | 1-2 | 1 | ([Han et al. 2006](#_ENREF_21)) |
| 15 | Q→K (sub) | likely benign | 1-2 | 1 |  |
| 24 | I→V (sub) | likely benign | 1-2 | 1 |  |
| 38-43 | RSIYHS→ - (del) | pathogenic | 1-2 | 5, 5, 1, 4, 1, 1 | ([Thiselton et al. 2002](#_ENREF_43)) |
| 80 | Y→C (sub) | pathogenic | 1-2 | 1 | ([Han et al. 2006](#_ENREF_21)) |
| 82 | Y→C (sub) | pathogenic | 1-2 | 1 |  |
| 95 | T→M (sub) | pathogenic | 1-2 | 2 | ([Ferre et al. 2009](#_ENREF_19)) |
| 102 | Y→C (sub) | pathogenic | 1-2 | 9 | ([Ferre et al. 2009](#_ENREF_19)) |
| 115 | A→V (sub) | pathogenic | 1-2 | 9 | ([Yu-Wai-Man et al. 2010b](#_ENREF_47)) |
| 127 | P→L (sub) | VUS | 3 | 9 |  |
| 158 | S→N (sub) | benign | 4 | 1 | ([Toomes et al. 2001](#_ENREF_44)) |
| 160 | E→Q (sub) | benign | 4 | 1 | ([Toomes et al. 2001](#_ENREF_44)) |
| 167 | P→L (sub) | benign | 4 | 9 | ([Thiselton et al. 2002](#_ENREF_43)) |
| 198 | D→N (sub) | VUS | 4b | 1 |  |
| 210 | A→V (sub) | benign | 5 | 1 | ([Pesch et al. 2001](#_ENREF_36)) |
| 247 | R→H (sub) | pathogenic | 5b | 4 | ([Cornille et al. 2008](#_ENREF_14)) |
| 256 | S→R (sub) | benign | 5b | 1 | ([Yu-Wai-Man et al. 2010b](#_ENREF_47)) |
| 277 | L→P (sub) | pathogenic | 6-8 | 8 | ([Ferre et al. 2009](#_ENREF_19)) |
| 290 | R→Q (sub) | VUS | 6-8 | 9 |  |
| 315 | L→F (sub) | pathogenic | 6-8 | 3 | ([Liskova et al. 2017](#_ENREF_29)) |
| 323 | Y→C (sub) | pathogenic | 6-8 | 9 |  |
| 324 | S→F (sub) | likely pathogenic | 6-8 | 9 |  |
| 325 | E→K (sub) | pathogenic | 6-8 | 9 | ([Pesch et al. 2001](#_ENREF_36)) |
| 327 | L→P (sub) | pathogenic | 6-8 | 9 | ([Baris et al. 2003](#_ENREF_7)) |
| 328 | D→A (sub) | pathogenic | 6-8 | 9 | ([Pesch et al. 2001](#_ENREF_36)) |
| 340 | Q→R (sub) | pathogenic | 6-8 | 6 | ([Yu-Wai-Man et al. 2010b](#_ENREF_47)) |
| 345 | R→Q (sub) | pathogenic | 6-8 | 9 | ([Alexander et al. 2000](#_ENREF_2); [Delettre et al. 2001](#_ENREF_16); [Pesch et al. 2001](#_ENREF_36); [Toomes et al. 2001](#_ENREF_44)) |
| 345 | R→W (sub) | pathogenic | 6-8 | 9 | ([Pesch et al. 2001](#_ENREF_36)) |
| 345 | R→L (sub) | pathogenic | 6-8 | 9 | ([Pesch et al. 2001](#_ENREF_36)) |
| 348-349 | VV→ - (del) | pathogenic | 9-16 (GTPase) | 9, 9 | ([Ferre et al. 2009](#_ENREF_19)) |
| 349 | V→ - (del) | pathogenic | 9-16 (GTPase) | 9 | ([Yu-Wai-Man et al. 2010a](#_ENREF_46)) |
| 351 | D→E (sub) | pathogenic | 9-16 (GTPase) | 9 | ([Ahmad et al. 2015](#_ENREF_1)) |
| 353 | S→N (sub) | likely pathogenic | 9-16 (GTPase) | 9 |  |
| 353 | S→G (sub) | pathogenic | 9-16 (GTPase) | 9 |  |
| 354 | A→P (sub) | pathogenic | 9-16 (GTPase) | 7 |  |
| 355 | G→E (sub) | pathogenic | 9-16 (GTPase) | 9 | ([Ban et al. 2010](#_ENREF_6); [Delettre et al. 2000](#_ENREF_17); [Toomes et al. 2001](#_ENREF_44)) |
| 357 | T→P (sub) | VUS | 9-16 (GTPase) | 9 |  |
| 358 | S→N (sub) | likely pathogenic | 9-16 (GTPase) | 9 | ([Gaier et al. 2017](#_ENREF_20)) |
| 358 | S→G (sub) | pathogenic | 9-16 (GTPase) | 9 | ([Chen et al. 2014](#_ENREF_10)) |
| 365 | Q→R (sub) | pathogenic | 9-16 (GTPase) | 9 | ([Ferre et al. 2009](#_ENREF_19)) |
| 368 | I→E (sub) | pathogenic | 9-16 (GTPase) | 9 | ([Cohn et al. 2007](#_ENREF_13)) |
| 377 | M→R (sub) | VUS | 9-16 (GTPase) | 9 |  |
| 379-381 | RSP→ - (del) | pathogenic | 9-16 (GTPase) | 9, 8, 9 | ([Puomila et al. 2005](#_ENREF_38)) |
| 384 | V→L (sub) | pathogenic | 9-16 (GTPase) | 9 | ([Iida et al. 2016](#_ENREF_24)) |
| 385 | T→S (sub) | pathogenic | 9-16 (GTPase) | 9 | ([Chen et al. 2013](#_ENREF_11)) |
| 392 | H→D (sub) | likely pathogenic | 9-16 (GTPase) | 9 |  |
| 394 | A→T (sub) | pathogenic | 9-16 (GTPase) | 9 | ([Rubegni et al. 2017](#_ENREF_39)) |
| 394 | A→D (sub) | VUS | 9-16 (GTPase) | 9 |  |
| 395 | L→P (sub) | VUS | 9-16 (GTPase) | 1 |  |
| 397 | K→E (sub) | pathogenic | 9-16 (GTPase) | 9 | ([Cohen et al. 2016](#_ENREF_12)) |
| 398 | D→A (sub) | pathogenic | 9-16 (GTPase) | 9 | ([Pretegiani et al. 2017](#_ENREF_37)) |
| 412 | A→T (sub) | pathogenic | 9-16 (GTPase) | 9 | ([Amati-Bonneau et al. 2008](#_ENREF_5); [Ferre et al. 2009](#_ENREF_19)) |
| 421 | R→Q (sub) | likely pathogenic | 9-16 (GTPase) | 9 |  |
| 431 | T→A (sub) | pathogenic | 9-16 (GTPase) | 9 | ([Cohn et al. 2007](#_ENREF_13)) |
| 432 | V→I (sub) | pathogenic | 9-16 (GTPase) | 9 | ([Chen et al. 2013](#_ENREF_11)) |
| 435 | E→D (sub) | pathogenic | 9-16 (GTPase) | 8 |  |
| 436 | T→S (sub) | pathogenic | 9-16 (GTPase) | 9 | ([Li et al. 2017](#_ENREF_27)) |
| 437 | I→M (sub) | pathogenic | 9-16 (GTPase) | 9 | ([Carelli et al. 2015](#_ENREF_9); [Ferre et al. 2009](#_ENREF_19); [Schaaf et al. 2011](#_ENREF_40)) |
| 439 | L→F (sub) | pathogenic | 9-16 (GTPase) | 9 | ([Toomes et al. 2001](#_ENREF_44)) |
| 450 | V→G (sub) | pathogenic | 9-16 (GTPase) | 9 | ([Li et al. 2017](#_ENREF_27)) |
| 451 | L→P (sub) | pathogenic | 9-16 (GTPase) | 9 | ([Ferre et al. 2009](#_ENREF_19)) |
| 451 | L→R (sub) | pathogenic | 9-16 (GTPase) | 9 | ([Thiselton et al. 2002](#_ENREF_43)) |
| 455 | P→A (sub) | pathogenic | 9-16 (GTPase) | 9 | ([Zhang et al. 2012](#_ENREF_48)) |
| 455 | P→L (sub) | pathogenic | 9-16 (GTPase) | 9 |  |
| 455 | P→S (sub) | pathogenic | 9-16 (GTPase) | 9 | ([Yu-Wai-Man et al. 2010a](#_ENREF_46)) |
| 455 | P→T (sub) | pathogenic | 9-16 (GTPase) | 9 | ([Li et al. 2017](#_ENREF_27)) |
| 456 | G→R (sub) | pathogenic | 9-16 (GTPase) | 9 | ([Chen et al. 2014](#_ENREF_10)) |
| 456 | G→D (sub) | pathogenic | 9-16 (GTPase) | 9 | ([Kim et al. 2005](#_ENREF_25)) |
| 457 | V→M (sub) | pathogenic | 9-16 (GTPase) | 9 | ([Bonneau et al. 2014](#_ENREF_8)) |
| 464 | G→D (sub) | VUS | 9-16 (GTPase) | 9 |  |
| 476 | I→II (dup) | pathogenic | 9-16 (GTPase) | 1 | ([Chen et al. 2014](#_ENREF_10)) |
| 477 | S→R (sub) | VUS | 9-16 (GTPase) | 9 |  |
| 481 | M→L (sub) | VUS | 9-16 (GTPase) | 9 |  |
| 483 | N→H (sub) | pathogenic | 9-16 (GTPase) | 9 |  |
| 484-485 | PN→ - (del) | pathogenic | 9-16 (GTPase) | 9, 9 | ([Ferre et al. 2009](#_ENREF_19)) |
| 485 | N→D (sub) | pathogenic | 9-16 (GTPase) | 9 | ([Ferre et al. 2009](#_ENREF_19)) |
| 487 | I→ - (del) | pathogenic | 9-16 (GTPase) | 9 | ([Alexander et al. 2000](#_ENREF_2); [Thiselton et al. 2002](#_ENREF_43)) |
| 488 | I→ - (del) | pathogenic | 9-16 (GTPase) | 9 | ([Alexander et al. 2000](#_ENREF_2)) |
| 489 | L→R (sub) | pathogenic | 9-16 (GTPase) | 9 | ([Chen et al. 2014](#_ENREF_10)) |
| 489 | L→P (sub) | pathogenic | 9-16 (GTPase) | 9 | ([Li et al. 2018](#_ENREF_26)) |
| 490 | C→R (sub) | pathogenic | 9-16 (GTPase) | 9 | ([Li et al. 2018](#_ENREF_26)) |
| 490 | C→Y (sub) | pathogenic | 9-16 (GTPase) | 9 | ([Chen et al. 2014](#_ENREF_10)) |
| 493 | D→V (sub) | pathogenic | 9-16 (GTPase) | 9 | ([Pesch et al. 2001](#_ENREF_36)) |
| 493 | D→A (sub) | likely pathogenic | 9-16 (GTPase) | 9 | ([Almind et al. 2012](#_ENREF_3)) |
| 493 | D→G (sub) | pathogenic | 9-16 (GTPase) | 9 | ([Dadgar et al. 2006](#_ENREF_15)) |
| 494 | G→V (sub) | pathogenic | 9-16 (GTPase) | 9 | ([Amati-Bonneau et al. 2008](#_ENREF_5); [Ban et al. 2010](#_ENREF_6); [Liguori et al. 2008](#_ENREF_28)) |
| 498 | A→T (sub) | pathogenic | 9-16 (GTPase) | 9 | ([Yu-Wai-Man et al. 2010b](#_ENREF_47)) |
| 499 | E→G (sub) | pathogenic | 9-16 (GTPase) | 9 | ([Gaier et al. 2017](#_ENREF_20)) |
| 500 | R→H (sub) | pathogenic | 9-16 (GTPase) | 9 | ([Amati-Bonneau et al. 2005](#_ENREF_4); [Amati-Bonneau et al. 2008](#_ENREF_5); [Ban et al. 2010](#_ENREF_6); [Payne et al. 2004](#_ENREF_35); [Shimizu et al. 2003](#_ENREF_41)) |
| 501 | S→C (sub) | VUS | 9-16 (GTPase) | 9 |  |
| 504 | T→R (sub) | pathogenic | 9-16 (GTPase) | 9 | ([Ferre et al. 2009](#_ENREF_19)) |
| 504 | T→P (sub) | pathogenic | 9-16 (GTPase) | 9 | ([Liskova et al. 2013](#_ENREF_30)) |
| 512 | P→T (sub) | VUS | 9-16 (GTPase) | 9 |  |
| 514 | G→E (sub) | likely pathogenic | 9-16 (GTPase) | 9 | ([Almind et al. 2012](#_ENREF_3)) |
| 517 | T→I (sub) | pathogenic | 9-16 (GTPase) | 9 |  |
| 518-519 | IF→IFIF (dup) | pathogenic | 9-16 (GTPase) | 9, 9 |  |
| 519 | F→ - (del) | pathogenic | 9-16 (GTPase) | 9 |  |
| 523 | K→E (sub) | pathogenic | 9-16 (GTPase) | 9 | ([Pesch et al. 2001](#_ENREF_36)) |
| 525 | D→G (sub) | pathogenic | 9-16 (GTPase) | 9 | ([Baris et al. 2003](#_ENREF_7)) |
| 526 | L→P (sub) | VUS | 9-16 (GTPase) | 9 |  |
| 537 | I→F (sub) | VUS | 9-16 (GTPase) | 9 |  |
| 542 | E→K (sub) | pathogenic | 9-16 (GTPase) | 9 | ([Bonneau et al. 2014](#_ENREF_8); [Ferre et al. 2009](#_ENREF_19)) |
| 543 | G→R (sub) | pathogenic | 9-16 (GTPase) | 9 | ([Yu-Wai-Man et al. 2010b](#_ENREF_47)) |
| 548 | M→K (sub) | VUS | 9-16 (GTPase) | 9 |  |
| 550 | A→V (sub) | pathogenic | 9-16 (GTPase) | 9 | ([Yu-Wai-Man et al. 2010b](#_ENREF_47)) |
| 556 | V→D (sub) | likely pathogenic | 9-16 (GTPase) | 9 |  |
| 558 | T→K (sub) | pathogenic | 9-16 (GTPase) | 9 | ([Thiselton et al. 2002](#_ENREF_43); [Toomes et al. 2001](#_ENREF_44)) |
| 560 | K→N (sub) | pathogenic | 9-16 (GTPase) | 6 | ([Toomes et al. 2001](#_ENREF_44)) |
| 576 | E→ - (del) | pathogenic | 9-16 (GTPase) | 1 | ([Ferre et al. 2009](#_ENREF_19)) |
| 589 | L→R (sub) | pathogenic | 17-18 | 9 | ([Spiegel et al. 2016](#_ENREF_42)) |
| 592-596 | HQVTT→P (del) | pathogenic | 17-18 | 8, 9, 9, 9, 9 | ([Li et al. 2017](#_ENREF_27)) |
| 600 | S→R (sub) | pathogenic | 17-18 | 9 | ([Amati-Bonneau et al. 2008](#_ENREF_5); [Ban et al. 2010](#_ENREF_6); [Ferre et al. 2009](#_ENREF_19); [Hudson et al. 2008](#_ENREF_23); [Nakamura et al. 2006](#_ENREF_32)) |
| 602 | A→AA (dup) | pathogenic | 17-18 | 9 | ([Kim et al. 2005](#_ENREF_25)) |
| 602 | A→E (sub) | pathogenic | 17-18 | 9 | ([Liskova et al. 2017](#_ENREF_29)) |
| 603 | V→I (sub) | pathogenic | 17-18 | 9 | ([Yu-Wai-Man et al. 2010b](#_ENREF_47)) |
| 605 | D→N (sub) | benign | 17-18 | 9 | ([Pesch et al. 2001](#_ENREF_36)) |
| 606 | C→ - (del) | pathogenic | 17-18 | 9 | ([Pesch et al. 2001](#_ENREF_36)) |
| 606 | C→Y (sub) | pathogenic | 17-18 | 9 | ([Ferre et al. 2009](#_ENREF_19); [Marelli et al. 2011](#_ENREF_31)) |
| 611 | V→E (sub) | pathogenic | 17-18 | 9 | ([Li et al. 2017](#_ENREF_27)) |
| 612 | R→P (sub) | pathogenic | 17-18 | 7 | ([Kim et al. 2005](#_ENREF_25)) |
| 626 | R→H (sub) | pathogenic | 17-18 | 9 | ([Thiselton et al. 2002](#_ENREF_43)) |
| 629 | L→P (sub) | pathogenic | 17-18 | 9 | ([Baris et al. 2003](#_ENREF_7)) |
| 637 | Y→C (sub) | pathogenic | 17-18 | 4 | ([Ferraris et al. 2008](#_ENREF_18)) |
| 641-644 | RELD→ - (del) | pathogenic | 17-18 | 9, 9, 9, 8 | ([Thiselton et al. 2002](#_ENREF_43)) |
| 645 | R→Q (sub) | pathogenic | 17-18 | 9 | ([Ferre et al. 2009](#_ENREF_19)) |
| 645 | R→W (sub) | pathogenic | 17-18 | 9 | ([Puomila et al. 2005](#_ENREF_38)) |
| 648 | L→P (sub) | pathogenic | 19-22 (Middle) | 9 | ([Ferre et al. 2009](#_ENREF_19)) |
| 648 | L→ - (del) | pathogenic | 19-22 (Middle) | 9 | ([Yen et al. 2010](#_ENREF_45)) |
| 656 | I→V (sub) | VUS | 19-22 (Middle) | 9 |  |
| 701 | S→L (sub) | pathogenic | 19-22 (Middle) | 6 | ([Ferre et al. 2009](#_ENREF_19)) |
| 707 | T→A (sub) | pathogenic | 19-22 (Middle) | 9 |  |
| 709 | D→G (sub) | VUS | 19-22 (Middle) | 9 |  |
| 738 | R→H (sub) | VUS | 19-22 (Middle) | 4 |  |
| 755-756 | LK→ - (del) | pathogenic | 19-22 (Middle) | 9, 9 | ([Baris et al. 2003](#_ENREF_7)) |
| 771 | D→E (sub) | benign | 19-22 (Middle) | 8 |  |
| 773 | A→E (sub) | pathogenic | 19-22 (Middle) | 9 | ([Chen et al. 2014](#_ENREF_10)) |
| 783 | N→K (sub) | pathogenic | 19-22 (Middle) | 9 | ([Ban et al. 2010](#_ENREF_6); [Puomila et al. 2005](#_ENREF_38)) |
| 785 | L→F (sub) | likely pathogenic | 19-22 (Middle) | 9 | ([Liskova et al. 2017](#_ENREF_29)) |
| 800 | I→M (sub) | pathogenic | 19-22 (Middle) | 9 | ([Li et al. 2018](#_ENREF_26)) |
| 823 | G→D (sub) | pathogenic | 23-25 (PH) | 9 | ([Ferre et al. 2009](#_ENREF_19)) |
| 825 | D→E (sub) | VUS | 23-25 (PH) | 3 |  |
| 836 | R→W (sub) | pathogenic | 23-25 (PH) | 7 | ([Ferre et al. 2009](#_ENREF_19)) |
| 838 | Q→R (sub) | pathogenic | 23-25 (PH) | 2 |  |
| 840 | Q→R (sub) | pathogenic | 23-25 (PH) | 8 | ([Ban et al. 2010](#_ENREF_6); [Delettre et al. 2001](#_ENREF_16); [Pesch et al. 2001](#_ENREF_36)) |
| 878 | S→Y (sub) | pathogenic | 23-25 (PH) | 3 | ([Ferre et al. 2009](#_ENREF_19)) |
| 886 | S→N (sub) | VUS | 23-25 (PH) | 1 |  |
| 887 | L→F (sub) | likely pathogenic | 23-25 (PH) | 4 | ([Almind et al. 2012](#_ENREF_3)) |
| 896 | Y→C (sub) | pathogenic | 23-25 (PH) | 4 | ([Han et al. 2006](#_ENREF_21)) |
| 898 | R→S (sub) | VUS | 23-25 (PH) | 7 |  |
| 933 | V→ - (del) | VUS | 26-28 (GED) | 9 |  |
| 937 | R→L (sub) | pathogenic | 26-28 (GED) | 9 | ([Ferre et al. 2009](#_ENREF_19)) |
| 937 | R→C (sub) | pathogenic | 26-28 (GED) | 9 | ([Rubegni et al. 2017](#_ENREF_39)) |
| 937 | R→G (sub) | VUS | 26-28 (GED) | 9 |  |
| 940 | R→H (sub) | pathogenic | 26-28 (GED) | 9 | ([Chen et al. 2014](#_ENREF_10)) |
| 942 | L→P (sub) | pathogenic | 26-28 (GED) | 9 | ([Ferre et al. 2009](#_ENREF_19)) |
| 958 | V→I (sub) | pathogenic | 26-28 (GED) | 9 | ([Li et al. 2017](#_ENREF_27)) |
| 962 | E→G (sub) | benign | 26-28 (GED) | 9 | ([Toomes et al. 2001](#_ENREF_44)) |
| 963-964 | KN→KIN (ins) | VUS | 26-28 (GED) | 9, 9 |  |
| 965 | V→ - (del) | pathogenic | 26-28 (GED) | 9 | ([Amati-Bonneau et al. 2008](#_ENREF_5); [Ban et al. 2010](#_ENREF_6)) |
| 965 | V→D (sub) | likely pathogenic | 26-28 (GED) | 9 | ([Almind et al. 2012](#_ENREF_3)) |
| 979 | K→ - (del) | pathogenic | 26-28 (GED) | 9 | ([Pretegiani et al. 2017](#_ENREF_37)) |
| 983 | L→P (sub) | pathogenic | 26-28 (GED) | 7 |  |
| 987 | R→C (sub) | pathogenic | 26-28 (GED) | 9 | ([Ferre et al. 2009](#_ENREF_19); [Nochez et al. 2009](#_ENREF_34)) |
| 987-989 | RVQ→ - (del) | pathogenic | 26-28 (GED) | 9, 9, 8 | ([Li et al. 2018](#_ENREF_26)) |
| 988 | V→F (sub) | likely pathogenic | 26-28 (GED) | 9 | ([Nasca et al. 2017](#_ENREF_33)) |
| 994 | L→P (sub) | pathogenic | 26-28 (GED) | 9 | ([Ban et al. 2010](#_ENREF_6); [Delettre et al. 2001](#_ENREF_16)) |
| 995-997 | KKV→I (del) | pathogenic | 26-28 (GED) | 1, 1, 5 | ([Zhang et al. 2014](#_ENREF_49)) |
| 1004 | L→P (sub) | pathogenic | 26-28 (GED) | 1 | ([Ferre et al. 2009](#_ENREF_19); [Yen et al. 2010](#_ENREF_45)) |
| 1004 | L→R (sub) | pathogenic | 26-28 (GED) | 1 | ([Chen et al. 2014](#_ENREF_10)) |
| 1004 | L→I (sub) | VUS | 26-28 (GED) | 1 |  |

^a^ The positions of the amino acid sites in human OPA1 protein are in accord with those of its transcript variant 8 (NP_570850.2).

^b^ sub: substitution; dup: duplication; del: deletion; ins: insertion.

^c^ pathogenic: disease associated; likely pathogenic: likely disease associated; VUS: variant of unknown significance; likely benign: likely not disease-associated; benign: not disease-associated.

^d^ Evolutionary conservation grade of the residues, analyzed by ConSurf (9, conserved; 1, variable).

**References**

Ahmad KE, Davis RL, and Sue CM. 2015. A novel OPA1 mutation causing variable age of onset autosomal dominant optic atrophy plus in an Australian family. *J Neurol* 262:2323-2328. 10.1007/s00415-015-7849-6

Alexander C, Votruba M, Pesch UE, Thiselton DL, Mayer S, Moore A, Rodriguez M, Kellner U, Leo-Kottler B, Auburger G, Bhattacharya SS, and Wissinger B. 2000. OPA1, encoding a dynamin-related GTPase, is mutated in autosomal dominant optic atrophy linked to chromosome 3q28. *Nat Genet* 26:211-215. 10.1038/79944

Almind GJ, Ek J, Rosenberg T, Eiberg H, Larsen M, Lucamp L, Brondum-Nielsen K, and Gronskov K. 2012. Dominant optic atrophy in Denmark - report of 15 novel mutations in OPA1, using a strategy with a detection rate of 90%. *BMC Med Genet* 13:65. 10.1186/1471-2350-13-65

Amati-Bonneau P, Guichet A, Olichon A, Chevrollier A, Viala F, Miot S, Ayuso C, Odent S, Arrouet C, Verny C, Calmels MN, Simard G, Belenguer P, Wang J, Puel JL, Hamel C, Malthiery Y, Bonneau D, Lenaers G, and Reynier P. 2005. OPA1 R445H mutation in optic atrophy associated with sensorineural deafness. *Ann Neurol* 58:958-963. 10.1002/ana.20681

Amati-Bonneau P, Valentino ML, Reynier P, Gallardo ME, Bornstein B, Boissiere A, Campos Y, Rivera H, de la Aleja JG, Carroccia R, Iommarini L, Labauge P, Figarella-Branger D, Marcorelles P, Furby A, Beauvais K, Letournel F, Liguori R, La Morgia C, Montagna P, Liguori M, Zanna C, Rugolo M, Cossarizza A, Wissinger B, Verny C, Schwarzenbacher R, Martin MA, Arenas J, Ayuso C, Garesse R, Lenaers G, Bonneau D, and Carelli V. 2008. OPA1 mutations induce mitochondrial DNA instability and optic atrophy 'plus' phenotypes. *Brain* 131:338-351. 10.1093/brain/awm298

Ban T, Heymann JA, Song Z, Hinshaw JE, and Chan DC. 2010. OPA1 disease alleles causing dominant optic atrophy have defects in cardiolipin-stimulated GTP hydrolysis and membrane tubulation. *Hum Mol Genet* 19:2113-2122. 10.1093/hmg/ddq088

Baris O, Delettre C, Amati-Bonneau P, Surget MO, Charlin JF, Catier A, Derieux L, Guyomard JL, Dollfus H, Jonveaux P, Ayuso C, Maumenee I, Lorenz B, Mohammed S, Tourmen Y, Bonneau D, Malthiery Y, Hamel C, and Reynier P. 2003. Fourteen novel OPA1 mutations in autosomal dominant optic atrophy including two de novo mutations in sporadic optic atrophy. *Hum Mutat* 21:656. 10.1002/humu.9152

Bonneau D, Colin E, Oca F, Ferre M, Chevrollier A, Gueguen N, Desquiret-Dumas V, N'Guyen S, Barth M, Zanlonghi X, Rio M, Desguerre I, Barnerias C, Momtchilova M, Rodriguez D, Slama A, Lenaers G, Procaccio V, Amati-Bonneau P, and Reynier P. 2014. Early-onset Behr syndrome due to compound heterozygous mutations in OPA1. *Brain* 137:e301. 10.1093/brain/awu184

Carelli V, Sabatelli M, Carrozzo R, Rizza T, Schimpf S, Wissinger B, Zanna C, Rugolo M, La Morgia C, Caporali L, Carbonelli M, Barboni P, Tonon C, Lodi R, and Bertini E. 2015. 'Behr syndrome' with OPA1 compound heterozygote mutations. *Brain* 138:e321. 10.1093/brain/awu234

Chen J, Xu K, Zhang X, Jiang F, Liu L, Dong B, Ren Y, and Li Y. 2014. Mutation screening of mitochondrial DNA as well as OPA1 and OPA3 in a Chinese cohort with suspected hereditary optic atrophy. *Invest Ophthalmol Vis Sci* 55:6987-6995. 10.1167/iovs.14-14953

Chen Y, Jia X, Wang P, Xiao X, Li S, Guo X, and Zhang Q. 2013. Mutation survey of the optic atrophy 1 gene in 193 Chinese families with suspected hereditary optic neuropathy. *Mol Vis* 19:292-302.

Cohen L, Tzur S, Goldenberg-Cohen N, Bormans C, Behar DM, and Reinstein E. 2016. Exome sequencing identified a novel de novo OPA1 mutation in a consanguineous family presenting with optic atrophy. *Genet Res (Camb)* 98:e10. 10.1017/S0016672316000070

Cohn AC, Toomes C, Potter C, Towns KV, Hewitt AW, Inglehearn CF, Craig JE, and Mackey DA. 2007. Autosomal dominant optic atrophy: penetrance and expressivity in patients with OPA1 mutations. *Am J Ophthalmol* 143:656-662. 10.1016/j.ajo.2006.12.038

Cornille K, Milea D, Amati-Bonneau P, Procaccio V, Zazoun L, Guillet V, El Achouri G, Delettre C, Gueguen N, Loiseau D, Muller A, Ferre M, Chevrollier A, Wallace DC, Bonneau D, Hamel C, Reynier P, and Lenaers G. 2008. Reversible optic neuropathy with OPA1 exon 5b mutation. *Ann Neurol* 63:667-671. 10.1002/ana.21376

Dadgar S, Hagens O, Dadgar SR, Haghighi EN, Schimpf S, Wissinger B, and Garshasbi M. 2006. Structural model of the OPA1 GTPase domain may explain the molecular consequences of a novel mutation in a family with autosomal dominant optic atrophy. *Exp Eye Res* 83:702-706. 10.1016/j.exer.2006.03.004

Delettre C, Griffoin JM, Kaplan J, Dollfus H, Lorenz B, Faivre L, Lenaers G, Belenguer P, and Hamel CP. 2001. Mutation spectrum and splicing variants in the OPA1 gene. *Hum Genet* 109:584-591. 10.1007/s00439-001-0633-y

Delettre C, Lenaers G, Griffoin JM, Gigarel N, Lorenzo C, Belenguer P, Pelloquin L, Grosgeorge J, Turc-Carel C, Perret E, Astarie-Dequeker C, Lasquellec L, Arnaud B, Ducommun B, Kaplan J, and Hamel CP. 2000. Nuclear gene OPA1, encoding a mitochondrial dynamin-related protein, is mutated in dominant optic atrophy. *Nat Genet* 26:207-210. 10.1038/79936

Ferraris S, Clark S, Garelli E, Davidzon G, Moore SA, Kardon RH, Bienstock RJ, Longley MJ, Mancuso M, Gutierrez Rios P, Hirano M, Copeland WC, and DiMauro S. 2008. Progressive external ophthalmoplegia and vision and hearing loss in a patient with mutations in POLG2 and OPA1. *Arch Neurol* 65:125-131. 10.1001/archneurol.2007.9

Ferre M, Bonneau D, Milea D, Chevrollier A, Verny C, Dollfus H, Ayuso C, Defoort S, Vignal C, Zanlonghi X, Charlin JF, Kaplan J, Odent S, Hamel CP, Procaccio V, Reynier P, and Amati-Bonneau P. 2009. Molecular screening of 980 cases of suspected hereditary optic neuropathy with a report on 77 novel OPA1 mutations. *Hum Mutat* 30:E692-705. 10.1002/humu.21025

Gaier ED, Boudreault K, Nakata I, Janessian M, Skidd P, DelBono E, Allen KF, Pasquale LR, Place E, Cestari DM, Stacy RC, Rizzo JF, 3rd, and Wiggs JL. 2017. Diagnostic genetic testing for patients with bilateral optic neuropathy and comparison of clinical features according to OPA1 mutation status. *Mol Vis* 23:548-560.

Han J, Thompson-Lowrey AJ, Reiss A, Mayorov V, Jia H, Biousse V, Newman NJ, and Brown MD. 2006. OPA1 mutations and mitochondrial DNA haplotypes in autosomal dominant optic atrophy. *Genet Med* 8:217-225. 10.109701.gim.0000214299.61930.c0

Hayashi T, Sasano H, Katagiri S, Tsunoda K, Kameya S, Nakazawa M, Iwata T, and Tsuneoka H. 2017. Heterozygous deletion of the OPA1 gene in patients with dominant optic atrophy. *Jpn J Ophthalmol* 61:395-401. 10.1007/s10384-017-0522-0

Hudson G, Amati-Bonneau P, Blakely EL, Stewart JD, He L, Schaefer AM, Griffiths PG, Ahlqvist K, Suomalainen A, Reynier P, McFarland R, Turnbull DM, Chinnery PF, and Taylor RW. 2008. Mutation of OPA1 causes dominant optic atrophy with external ophthalmoplegia, ataxia, deafness and multiple mitochondrial DNA deletions: a novel disorder of mtDNA maintenance. *Brain* 131:329-337. 10.1093/brain/awm272

Iida K, Ohkuma Y, Hayashi T, Katagiri S, Fujita T, Tsunoda K, Yamada H, and Tsuneoka H. 2016. A novel heterozygous splice site OPA1 mutation causes exon 10 skipping in Japanese patients with dominant optic atrophy. *Ophthalmic Genet* 37:354-356. 10.3109/13816810.2015.1066829

Kim JY, Hwang JM, Ko HS, Seong MW, Park BJ, and Park SS. 2005. Mitochondrial DNA content is decreased in autosomal dominant optic atrophy. *Neurology* 64:966-972. 10.1212/01.WNL.0000157282.76715.B1

Li H, Jones EM, Li H, Yang L, Sun Z, Yuan Z, Chen R, Dong F, and Sui R. 2018. Clinical and genetic features of eight Chinese autosomal-dominant optic atrophy pedigrees with six novel OPA1 pathogenic variants. *Ophthalmic Genet* 39:569-576. 10.1080/13816810.2018.1466337

Li Y, Li J, Jia X, Xiao X, Li S, and Guo X. 2017. Genetic and Clinical Analyses of DOA and LHON in 304 Chinese Patients with Suspected Childhood-Onset Hereditary Optic Neuropathy. *PLoS One* 12:e0170090. 10.1371/journal.pone.0170090

Liguori M, La Russa A, Manna I, Andreoli V, Caracciolo M, Spadafora P, Cittadella R, and Quattrone A. 2008. A phenotypic variation of dominant optic atrophy and deafness (ADOAD) due to a novel OPA1 mutation. *J Neurol* 255:127-129. 10.1007/s00415-008-0571-x

Liskova P, Tesarova M, Dudakova L, Svecova S, Kolarova H, Honzik T, Seto S, and Votruba M. 2017. OPA1 analysis in an international series of probands with bilateral optic atrophy. *Acta Ophthalmol* 95:363-369. 10.1111/aos.13285

Liskova P, Ulmanova O, Tesina P, Melsova H, Diblik P, Hansikova H, Tesarova M, and Votruba M. 2013. Novel OPA1 missense mutation in a family with optic atrophy and severe widespread neurological disorder. *Acta Ophthalmol* 91:e225-231. 10.1111/aos.12038

Marelli C, Amati-Bonneau P, Reynier P, Layet V, Layet A, Stevanin G, Brissaud E, Bonneau D, Durr A, and Brice A. 2011. Heterozygous OPA1 mutations in Behr syndrome. *Brain* 134:e169; author reply e170. 10.1093/brain/awq306

Nakamura M, Lin J, Ueno S, Asaoka R, Hirai T, Hotta Y, Miyake Y, and Terasaki H. 2006. Novel mutations in the OPA1 gene and associated clinical features in Japanese patients with optic atrophy. *Ophthalmology* 113:483-488 e481. 10.1016/j.ophtha.2005.10.054

Nasca A, Rizza T, Doimo M, Legati A, Ciolfi A, Diodato D, Calderan C, Carrara G, Lamantea E, Aiello C, Di Nottia M, Niceta M, Lamperti C, Ardissone A, Bianchi-Marzoli S, Iarossi G, Bertini E, Moroni I, Tartaglia M, Salviati L, Carrozzo R, and Ghezzi D. 2017. Not only dominant, not only optic atrophy: expanding the clinical spectrum associated with OPA1 mutations. *Orphanet J Rare Dis* 12:89. 10.1186/s13023-017-0641-1

Nochez Y, Arsene S, Gueguen N, Chevrollier A, Ferre M, Guillet V, Desquiret V, Toutain A, Bonneau D, Procaccio V, Amati-Bonneau P, Pisella PJ, and Reynier P. 2009. Acute and late-onset optic atrophy due to a novel OPA1 mutation leading to a mitochondrial coupling defect. *Mol Vis* 15:598-608.

Payne M, Yang Z, Katz BJ, Warner JE, Weight CJ, Zhao Y, Pearson ED, Treft RL, Hillman T, Kennedy RJ, Meire FM, and Zhang K. 2004. Dominant optic atrophy, sensorineural hearing loss, ptosis, and ophthalmoplegia: a syndrome caused by a missense mutation in OPA1. *Am J Ophthalmol* 138:749-755. 10.1016/j.ajo.2004.06.011

Pesch UE, Leo-Kottler B, Mayer S, Jurklies B, Kellner U, Apfelstedt-Sylla E, Zrenner E, Alexander C, and Wissinger B. 2001. OPA1 mutations in patients with autosomal dominant optic atrophy and evidence for semi-dominant inheritance. *Hum Mol Genet* 10:1359-1368.

Pretegiani E, Rosini F, Rufa A, Gallus GN, Cardaioli E, Da Pozzo P, Bianchi S, Serchi V, Collura M, Franceschini R, Bianchi Marzoli S, Dotti MT, and Federico A. 2017. Genotype-phenotype and OCT correlations in Autosomal Dominant Optic Atrophy related to OPA1 gene mutations: Report of 13 Italian families. *J Neurol Sci* 382:29-35. 10.1016/j.jns.2017.09.018

Puomila A, Huoponen K, Mantyjarvi M, Hamalainen P, Paananen R, Sankila EM, Savontaus ML, Somer M, and Nikoskelainen E. 2005. Dominant optic atrophy: correlation between clinical and molecular genetic studies. *Acta Ophthalmol Scand* 83:337-346. 10.1111/j.1600-0420.2005.00448.x

Rubegni A, Pisano T, Bacci G, Tessa A, Battini R, Procopio E, Giglio S, Pasquariello R, Santorelli FM, Guerrini R, and Nesti C. 2017. Leigh-like neuroimaging features associated with new biallelic mutations in OPA1. *Eur J Paediatr Neurol* 21:671-677. 10.1016/j.ejpn.2017.04.004

Schaaf CP, Blazo M, Lewis RA, Tonini RE, Takei H, Wang J, Wong LJ, and Scaglia F. 2011. Early-onset severe neuromuscular phenotype associated with compound heterozygosity for OPA1 mutations. *Mol Genet Metab* 103:383-387. 10.1016/j.ymgme.2011.04.018

Shimizu S, Mori N, Kishi M, Sugata H, Tsuda A, and Kubota N. 2003. A novel mutation in the OPA1 gene in a Japanese patient with optic atrophy. *Am J Ophthalmol* 135:256-257.

Spiegel R, Saada A, Flannery PJ, Burte F, Soiferman D, Khayat M, Eisner V, Vladovski E, Taylor RW, Bindoff LA, Shaag A, Mandel H, Schuler-Furman O, Shalev SA, Elpeleg O, and Yu-Wai-Man P. 2016. Fatal infantile mitochondrial encephalomyopathy, hypertrophic cardiomyopathy and optic atrophy associated with a homozygous OPA1 mutation. *J Med Genet* 53:127-131. 10.1136/jmedgenet-2015-103361

Thiselton DL, Alexander C, Taanman JW, Brooks S, Rosenberg T, Eiberg H, Andreasson S, Van Regemorter N, Munier FL, Moore AT, Bhattacharya SS, and Votruba M. 2002. A comprehensive survey of mutations in the OPA1 gene in patients with autosomal dominant optic atrophy. *Invest Ophthalmol Vis Sci* 43:1715-1724.

Toomes C, Marchbank NJ, Mackey DA, Craig JE, Newbury-Ecob RA, Bennett CP, Vize CJ, Desai SP, Black GC, Patel N, Teimory M, Markham AF, Inglehearn CF, and Churchill AJ. 2001. Spectrum, frequency and penetrance of OPA1 mutations in dominant optic atrophy. *Hum Mol Genet* 10:1369-1378.

Yen MY, Wang AG, Lin YC, Fann MJ, and Hsiao KJ. 2010. Novel mutations of the OPA1 gene in Chinese dominant optic atrophy. *Ophthalmology* 117:392-396 e391. 10.1016/j.ophtha.2009.07.019

Yu-Wai-Man P, Griffiths PG, Burke A, Sellar PW, Clarke MP, Gnanaraj L, Ah-Kine D, Hudson G, Czermin B, Taylor RW, Horvath R, and Chinnery PF. 2010a. The prevalence and natural history of dominant optic atrophy due to OPA1 mutations. *Ophthalmology* 117:1538-1546, 1546 e1531. 10.1016/j.ophtha.2009.12.038

Yu-Wai-Man P, Griffiths PG, Gorman GS, Lourenco CM, Wright AF, Auer-Grumbach M, Toscano A, Musumeci O, Valentino ML, Caporali L, Lamperti C, Tallaksen CM, Duffey P, Miller J, Whittaker RG, Baker MR, Jackson MJ, Clarke MP, Dhillon B, Czermin B, Stewart JD, Hudson G, Reynier P, Bonneau D, Marques W, Jr., Lenaers G, McFarland R, Taylor RW, Turnbull DM, Votruba M, Zeviani M, Carelli V, Bindoff LA, Horvath R, Amati-Bonneau P, and Chinnery PF. 2010b. Multi-system neurological disease is common in patients with OPA1 mutations. *Brain* 133:771-786. 10.1093/brain/awq007

Zhang J, Yuan Y, Lin B, Feng H, Li Y, Dai X, Zhou H, Dong X, Liu XL, and Guan MX. 2012. A novel OPA1 mutation in a Chinese family with autosomal dominant optic atrophy. *Biochem Biophys Res Commun* 419:670-675. 10.1016/j.bbrc.2012.02.073

Zhang L, Shi W, Song L, Zhang X, Cheng L, Wang Y, Ge X, Li W, Zhang W, Min Q, Jin ZB, Qu J, and Gu F. 2014. A recurrent deletion mutation in OPA1 causes autosomal dominant optic atrophy in a Chinese family. *Sci Rep* 4:6936. 10.1038/srep06936
